# A Low-Cost Water Depth and Electrical Conductivity Sensor for Detecting Inputs into Urban Stormwater Networks

**DOI:** 10.3390/s21093056

**Published:** 2021-04-27

**Authors:** Baiqian Shi, Stephen Catsamas, Peter Kolotelo, Miao Wang, Anna Lintern, Dusan Jovanovic, Peter M. Bach, Ana Deletic, David T. McCarthy

**Affiliations:** 1 BoSL Water Monitoring and Control, Department of Civil Engineering, Monash University, Wellington Road, Clayton 3800, Australia; baiqian.shi1@monash.edu (B.S.); stephen.catsamas@monash.edu (S.C.); Peter.Kolotelo@monash.edu (P.K.); miao.wang@monash.edu (M.W.); anna.lintern@monash.edu (A.L.); dusan.jovanovic@gf.uns.ac.rs (D.J.); 2 Faculty of Civil Engineering, University of Novi Sad, Kozaračka 2a, 24000 Subotica, Serbia; 3 Swiss Federal Institute of Aquatic Science & Technology (Eawag), 8600 Dübendorf, Switzerland; peter.bach@eawag.ch; 4 Institute of Environmental Engineering, ETH Zürich, 8093 Zürich, Switzerland; 5 School of Civil and Environmental Engineering, Faculty of Engineering, Queensland University of Technology, GPO Box 2434, Brisbane 4001, Australia; ana.deletic@qut.edu.au

**Keywords:** water level measurement, electric conductivity, real-time environmental monitoring, illegal discharge detection, distributed sensing, low cost, low power, water IoT

## Abstract

High-resolution data collection of the urban stormwater network is crucial for future asset management and illicit discharge detection, but often too expensive as sensors and ongoing frequent maintenance works are not affordable. We developed an integrated water depth, electrical conductivity (EC), and temperature sensor that is inexpensive (USD 25), low power, and easily implemented in urban drainage networks. Our low-cost sensor reliably measures the rate-of-change of water level without any re-calibration by comparing with industry-standard instruments such as HACH and HORIBA’s probes. To overcome the observed drift of level sensors, we developed an automated re-calibration approach, which significantly improved its accuracy. For applications like monitoring stormwater drains, such an approach will make higher-resolution sensing feasible from the budget control considerations, since the regular sensor re-calibration will no longer be required. For other applications like monitoring wetlands or wastewater networks, a manual re-calibration every two weeks is required to limit the sensor’s inaccuracies to ±10 mm. Apart from only being used as a calibrator for the level sensor, the conductivity sensor in this study adequately monitored EC between 0 and 10 mS/cm with a 17% relative uncertainty, which is sufficient for stormwater monitoring, especially for real-time detection of poor stormwater quality inputs. Overall, our proposed sensor can be rapidly and densely deployed in the urban drainage network for revolutionised high-density monitoring that cannot be achieved before with high-end loggers and sensors.

## 1. Introduction

Stormwater runoff from urbanised areas contains significant levels of pollutants [1,2], which requires continuous monitoring of the stormwater network to understand their magnitude and impact on downstream ecosystems. Physical parameters including water level, temperature, and electrical conductivity (EC), together with other chemical and microbiological parameters are usually used for this purpose [3]. Conventionally, local authorities establish monitoring stations along the main drainage reaches to provide essential data that can be used to describe general waterway health. However, many researchers have emphasised that higher spatial and temporal resolution data should be collected in future via a smart water system to better understand network dynamics, to improve stormwater modelling [4,5], and to even support the existing stormwater infrastructure management in overcoming the challenges imposed by climate change and rapid urbanisation [6]. More urgently, illicit discharges such as cross-connection with the wastewater system, industrial discharge, and spills are considered as a major pollution source in our stormwater system [7,8]. However, conventional detection methods, including visual inspection, dye testing, and CCTV (closed-circuit television) cameras are not necessarily efficient and effective enough to locate and eliminate most point discharge sources in an urban catchment [9,10]. This is because these measures usually require substantial on-site work and human and financial resources to conduct (i.e., manually searching a vast urban catchment or conducting camera real-time data check), thereby making high spatial and temporal resolution monitoring in near-real-time cumbersome and costly. To confirm the severity of a detected illicit discharge source, a grab sample is usually collected to analyse various indicative parameters, including ammonia, conductivity, and microbial parameters [11]. However, effective grab sampling requires field crews to be at the right location at the right time, which makes the sample hard to be collected and results in low work efficiency. Furthermore, long lab turnover times on sample analyses will not allow investigators to receive the result before the end of an intermittent or transitory discharge event [10,12]. Hence, for illicit discharge detection, there is a need to monitor both water level and other quality parameters in real-time distributed network to eliminate the illegal behaviours from happening.

Traditionally, industrial-standard instruments such as the HACH submerged pressure and AV sensor [13] and HORIBA water quality meters [14] are used for the water monitoring, which provides users with reliable and accurate readings of water level and other quality parameters over time. However, their relatively high cost, bulky equipment (monitoring boxes, batteries, solar panels, and loggers) and the effort of installation and maintenance (i.e., confined space entry into the underground drain) limit their widespread use in distributed urban stormwater monitoring networks. This emphasised that, to make running such a sensor network affordable, future sensor technology must be not only low-cost, but also low power, easy to install and less maintenance required to make. For example, instead of installing multiple sensors at one location, the future low-cost sensors can combine water quantity with quality probes into one design to minimise the complexity of installation, which is exactly the design philosophy of this study.

This study was inspired by the recent developments of Arduino-based sensors and the Internet-of-Things (IoT) concept for environmental monitoring [15,16,17,18]. In particular, the aim of this study was to develop a low-cost sensor that can be used for real-time stormwater monitoring and illegal discharge detection purposes by concurrently monitoring water depths, electrical conductivities, and temperatures with good accuracies. The objectives of this paper are threefold: (1) test a newly developed integrated sensor to understand its performance; (2) validate the sensor’s durability under the hash stormwater network environment; and (3) estimate the uncertainty of the sensor for measuring both absolute readings and the trend over time. Through this study, we developed the first sensor that can simultaneously measure electrical conductivity, temperature, and water depth. This sensor can automatically adjust itself for drifting errors, which significantly reduce the ongoing sensor maintenance requirements. It will revolutionise the way how stormwater systems are monitored, resulting in high spatial resolution datasets that will inform better management options. Further, these sensors will have ready adoption pathways into other water systems, such as wastewater and natural waterways.

## 2. Sensor Development

### 2.1. Measured Parameters and Existing Research

The developed sensor in this study was designed to measure water levels, electric conductivities, and temperature. The water level is an essential parameter of stormwater monitoring and a direct indicator of dry weather discharges. On top of the depth, other water quality parameters should also be monitored at the same time to help identify the source of discharges [11]. Among all parameters, temperature and conductivity have been used in the past to successfully distinguish wastewater or industrial discharges from dry weather stormwater baseflow [10]. This is because domestic wastewater’s temperature is significantly higher than that of groundwater [19], and the water conductivity is highly correlated with nitrate, phosphorous, and dissolved metals which usually link to specific industrial activities such as metal plating [7,10,11]. Instead of only analysing the absolute level of water level, temperature, and EC, the general patterns or rather the rates of change are also considered as strong indicators of illicit discharges [20,21].

There are three common technical approaches for water depth sensing including ultrasonic-based distance measuring, capacitive-based sensing, and traditional water pressure monitoring. The ultrasonic sensor, such as the Arduino compatible sensor HC-SR04 has been recognised as a cheap solution of water level sensing, which can be easily installed on top of the target waterbody such as an open water surface or a rainwater tank [22]. More recently, the continuous-wave Doppler radar sensor was introduced to achieve millimetre-level accuracy of water level sensing [23] and has been trialled in urban cities for inundation level detection [24]. However, the ultrasonic/radar sensing approach is less viable within the urban stormwater context due to: (1) unevenness of concrete walls inside stormwater manhole might, which affect sensor performance due to its wide detection angle; (2) debris carried by flow in urban drains might increase the sensor’s uncertainty; and (3) installation and calibration times of the ultrasonic sensor at each site are too lengthy. 

Capacitive-type water level sensors have been developed based on the generation of fringing fields between positive and negative electrodes—as water level increases, higher proportions of the capacitive sensor will be submerged in water, which increases the electrode capacitance level [25,26]. Researchers provided different design solutions, such as using an interdigital capacitive sensor or a polyethylene pipe to successfully measure water level [25,26]. Such methods are suitable for large water storage tanks by attaching the sensor along the inside wall of the tank. However, due to the irregular and unidentical shape of urban stormwater drains and outfalls, the capacitive-type sensor might not be an ideal low-cost solution.

Perhaps the most traditional approach to water level sensing is by measuring water pressure using a submerged probe. New off-the-shelf technologies have lowered pressure measurement costs, including the MS5837-30BA from TE Connectivity (Schaffhausen, Switzerland) [27]. As adequate detection of pollution sources also requires water quality measurement (e.g., water temperature and electrical conductivity), underwater pressure sensors are also more promising compared with the other two types of sensors discussed above as they remain out of the water column and hence separate sensors would be required. To the authors’ knowledge, no researchers have, however, attempted to use the cheap pressure sensors in any IoT based distributed water monitoring system and, hence, their performance under different installation conditions need to be further investigated. 

Recently, many low-cost electric conductivity sensors have also been developed. Parra et al. [28] used two coils to generate and detect the magnetic field, which achieved a detection range between 0.4 and 76 mS/cm with an error of 20–30%. Bur for urban stormwater monitoring purpose, the proposed coil sensor’s size will be a limitation as the sensor needs to be fully submerged underwater. In addition, based on the voltage divider theory, Carminati and Luzzatto-Fegiz [29] modified the micro-USB cable to successfully measure the water EC between 1 and 150 mS/cm. These studies suggest that in order to measure the conductivity of water, a sensor can be quickly developed with easy-accessible materials. However, the cost of a conductivity sensor is still not low enough for high spatial distribution monitoring.

### 2.2. Depth and Temperature Sensors

**Figure 1:**
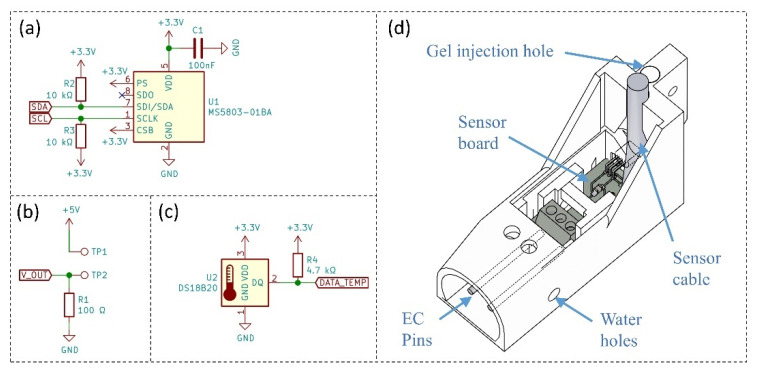
(**a**) The circuit design of MS5803-01BA sensor with I^2^C communication: +3.3 V for the power supply, GND for ground connection, and SDA and SCL for the I^2^C bus; (**b**) a simple voltage divider circuit to measure the conductivity of water-based on 5 V power supply: R1 is the selected 100 Ω resistor in this study, TP1 and TP2 are the two screw terminals on the PCB circuit to connect with the stainless steel rod, and V_OUT connects to one of the analog pins on the MCU to measure the voltage drop across R1; (**c**) the circuit diagram of a digital temperature sensor DS18B20: DATA_TEMP wire connects to one of the digital pins on the MCU to measure the environment temperature; and (**d**) sensor housing design with a vertical wall at the back of the sensor for easier installation.

To overcome the drawbacks of existing low-cost techniques and successfully monitor the water depth of stormwater, a high-resolution I^2^C based absolute pressure and temperature sensor MS5803-01BA from TE Connectivity [30] is selected in this study (Figure 1a; the design presented in this study are openly available in [Water depth, electrical conductivity and temperature sensor design] at [10.17632/kf45vdpdnh.2], published on: 19 March 2021). The depth and temperature sensor requires four-wire connections including 3.3 V power supply, ground, SDA and SCL, which can be easily connected with popular microcontroller units (MCU) such as Arduino Uno and Raspberry Pi. Since MS5803 is an absolute pressure sensor that measures the gauged water pressure plus the atmospheric pressure, another MS5803-01BA sensor is required to place in air to account for the changing air pressure over time; we propose the installation of an air pressure sensor within a 1 km radius of each depth sensor, but multiple depth sensors can rely on a single air pressure sensor. Water depth is then calculated based on the hydrostatic pressure equation (Equation (1)). While the MS5803-01BA has an integrated temperature sensor, its responsiveness was limited by the gel used to seal the device and hence we introduced another digital temperature sensor DS18B20 (see Figure 1c), to ensure accurate and responsive temperature measurements (±0.5 °C [31]). Reliable temperature sensing is also crucial for correctly calculating the water level and conductivity.

A simple one-point calibration is required to calculate the calibration offset (in Equation (1)) before sensor deployment to correct for the inherent difference between the air and the depth MS5803 sensors. The calibration offset is the difference between two sensors when they are both placed in air.

(1) $$d=\frac{p_{abs}-p_{air}-e_{cal}}{\rho_{water}\times g}$$

where *d* is water depth (m), *p_abs_* is the absolute pressure (mbar), *p_air_* is the ambient air pressure (mbar), *e_cal_* is the calibration offset (mbar) and *ρ* is the density of water at a specific temperature (kg/m^3^), and *g* is the gravitational acceleration constant (9.81 m/s^2^).

### 2.3. Electrical Conductivity Sensor

The electric conductivity sensor in this study adopts the concept of a simple voltage divider; similar work has been done by Ratcliffe [32] by using a USD 3 power cable. As shown in Figure 1b, two screw terminal pins (TP1 and TP2) are soldered on the PCB and facing the front to hold two stainless-steel rods tight. V_OUT wire connects to one Analog pin on the MCU to measure the voltage drop across the 100 Ω resistor. When the sensor is in air without contacting any liquid, the voltage drop across the constant resistor will be 0 V as it is an open circuit. When the sensor is in contact with liquid, the resistance *R_EC_* across TP1 and TP2 is linked to the EC level of water, thereby affecting the voltage drop across the resistor. The conductivity (*EC_T_*) of water between TP1 and TP2 can be then calculated based on the reciprocal relationship between the resistance and conductance as shown below in Equation (2):

(2) $$EC_{T}=k\times G=k\times \frac{1}{R_{EC}}$$

where *EC_T_* is the electrical conductivity at the temperature of the time of measurement. *G* is the conductance of the sensor at the temperature (t) when the measurement is taken, *k* is the cell constant (directly proportional to the distance separating the two conductive pins and inversely proportional to their surface area; for this sensor, it is ~0.84). To report the EC at 25 °C as a standard unit of environmental water monitoring, the effect of temperature on the EC needs to be corrected based on Equation (3) shown below [33]:

(3) $$EC_{25℃}=\frac{EC_{T}}{\left[1+0.019\times \left(T-25\right)\right]}$$

where *EC*_25__°C_ represents the electric conductivity at 25 °C and t is the liquid temperature at the time of measurement. The EC sensor needs to be calibrated with standard solutions before field real-time monitoring deployments to achieve the best sensor performance and resolution for a specific measuring range.

### 2.4. Structural Design

The sensor housing is specifically designed for easy installation in the urban drainage pipe without doing confined space entry (see Figure 1d). The current design introduced a vertical wall at the back of the sensor case, which is used to connect with a stainless-steel pole (bolted through). This allows the sensor to be secured at the invert of the stormwater drain during large flow events by fixing the pole on the concrete wall.

**Figure A1:**
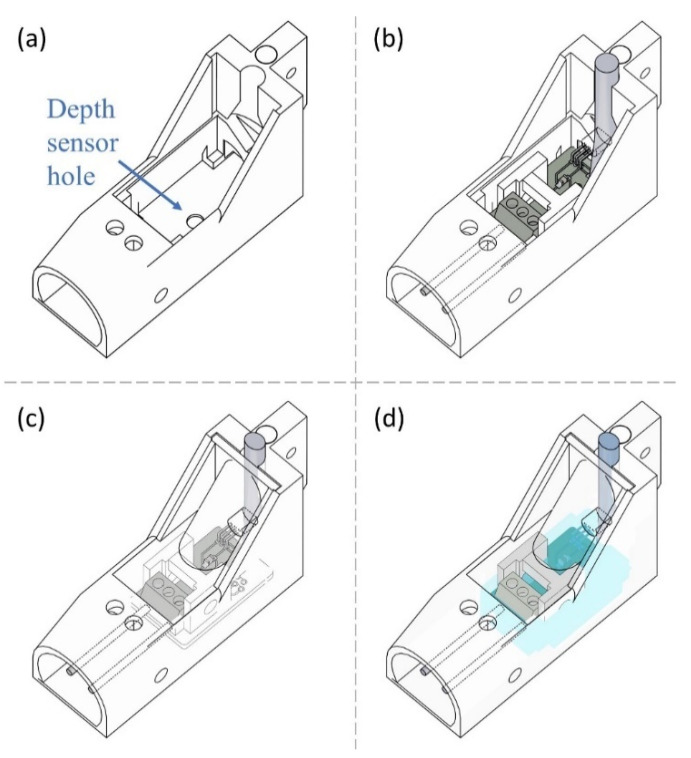
(**a**) The base on the designed 3D sensor covers for easy and quick installation in the field; (**b**) how the sensor board, EC pins, and the sensor holder are placed into the sensor case; (**c**) the sensor case lid to push the sensor board down and forms as physical protection of the sensor; and (**d**) injecting the potting compound gel for waterproofing purpose.

The sensor housing is currently 3D-printed by using the PET-G filament due to its higher strength and watertightness compared with other commonly used materials such as ABS and PLA (3D print design and STL files are available on the Source Repository: 10.17632/kf45vdpdnh.1). An insulating polymeric gel is utilised to provide the sensor with IP68 level of waterproof protection (see Figure A1). The 3D-printed sensor housing is designed to fill the potting compound from the bottom of the case, which ensures the gel will reach all corners on all sides of the sensor. The total cost of the proposed all-in-one sensor is roughly USD 25, including all components (a components list is available on the [Water depth, electrical conductivity and temperature sensor design] at [10.17632/kf45vdpdnh.2], published on: 19 March 2021).

## 3. Material and Methods

### 3.1. Depth Sensor

#### 3.1.1. Laboratory Water Column Test

**Figure 2:**
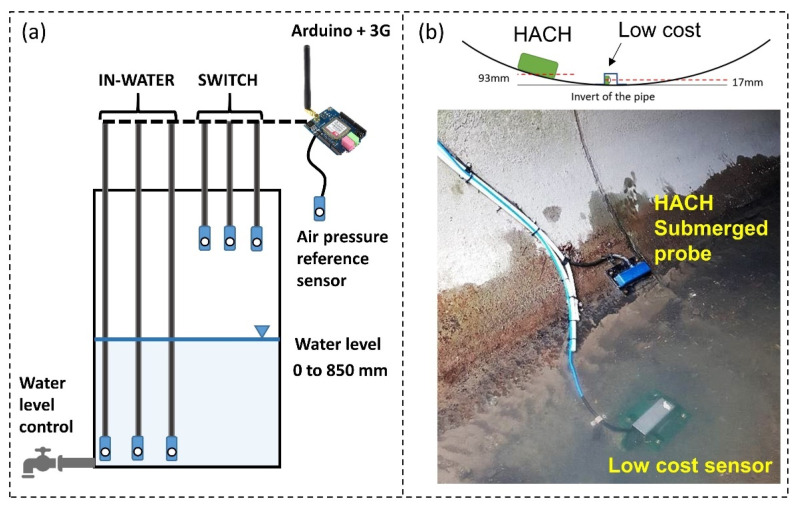
(**a**) Lab experiment setup in a water column for testing the proposed water depth sensor: water level is controlled through a tap and the level was changed between 0 and 850 mm; (**b**) Field installation layout to compare the performance between the low-cost and HACH submerged probe; the HACH sensor is 93 mm above the invert of the pipe and the low-cost sensor is 17 mm above the invert.

The developed low-cost water depth sensor was tested in a lab water column for two months to understand the performance of the sensor (Figure 2a). To assess the impact of continually inundating the sensors as compared to the wetting and drying of the sensors (common in flashy stormwater catchments), six sensors were placed in the column with three of them always fixed at the column’s base (IN-WATER 1–3: simulating open waterbodies like a lake or a pond), and another three switching between being in the water and in air over the testing period (SWITCH 1–3: simulating stormwater drains during dry and wet periods). An air reference sensor was placed just next to the laboratory setup. Sensor data was recorded at 6 min intervals by uploading the data to a web server. All the tested sensors were calibrated on the first day of this experiment, using the processes explained above.

The true water level was recorded manually throughout the experiment and these manual measurements were directly correlated with the low-cost sensor data collected at the same time to demonstrate the sensor performance (*n* = 21 data points). While this comparison of 21 datapoints was useful, more than 7500 water level measurements were made by the low-cost sensor during the testing period. To make use of these extra data points, we linearly interpolate our manual datasets; this is justified since evaporation rates were very small (<5 mm difference between any two manual measurement dates) and were found to be very constant (inside, constant temperature, no wind laboratory). Using this interpolated data, the absolute depth error was estimated for each of the sensors’ depth measurements:

(4) $$d_{err}=d_{sensor}-d_{true}$$

where *d_err_* is the absolute difference between the sensor measurement *d_sensor_* and the manually measured and linearly interpolated depth measurement *d_true_*. This was then used to estimate the percentage error (%) for each measurement:

(5) $$d_{err\%}=\frac{\left|d_{err}\right|}{d_{true}}$$

The relative uncertainty *u(d_sensor_)* of the proposed low-cost depth sensor is then reported by averaging the percentage error over all data points *N*:

(6) $$u\left(d_{sensor}\right)=\frac{\sum_{i=1}^{N}\left(d_{err\%}\right)}{N}$$

#### 3.1.2. In Situ Comparison between Low-Cost and a High-End Sensor

The low-cost depth sensor was tested in the field for almost a year (29 March 2019 and 4 February 2020). Our first low-cost depth sensor (Sensor 1) failed after two month of deployment due to water ingress. Our second sensor (Sensor 2), with improved waterproofing as presented herein, was in operation at the same position with the same installation method from early July 2019 until February 2020.

**Sensor installation.** To examine the performance and reliability of the new sensor, it was installed adjacent to a HACH submerged probe in a drainage pipe with open access (Figure 2b). The HACH probe was installed towards the side and 93 mm above the invert of the pipe due to the high sediment accumulation, which always buries the sensor, thereby affecting its accuracy. Our low-cost depth sensor was not impacted by sediment loads and, hence, was attached to a stainless-steel cover and screwed down to the bottom of the pipe (17 mm above the invert). This complex installation method was done only to accurately compare the low-cost sensor performance with the high-end instruction and is not anticipated to be the typical method of installing for the low-cost sensor design in this study due to potentially high labour cost and confined space entry. Accounting for the level differences between low-cost sensor, the HACH probe and the invert of the pipe, true water levels above the pipe’s invert are reported in this study, meaning that the minimum depth recorded by our low-cost sensor was 17 mm and the minimum depth recorded by the HACH was 93 mm.

The air reference sensor for this field test was placed in a cabinet just 10 m away from the sampling point. Both Sensor 1 and 2 were calibrated with the air reference sensor on the day of installation. Their calibration offsets were 2.35 mbar and 1.63 mbar, respectively.

**Sensor program and adjustments.** The low-cost depth sensor is programmed to scan water pressure six times every minute with a 10s scanning interval to obtain minute-averaged water pressure. To save battery life, the data logger did not upload each minute’s reading to a web-based data server. Instead, a variable logging interval ranging between a minute to an hour was utilised. The data uploading was triggered when the water pressure changed more than 1 mbar (roughly 10 mm) when compared with the data of a minute ago. The maximum logging interval was set to be one hour (for dry weather period—The water pressure will be logged every hour even the water level is constant).

When calculating water depth from Equation (1), air reference pressure needs to be measured roughly at the same time as water pressure. As such, we logged air pressure every minute to ensure this data was always available when a depth measurement was made. All data manipulations, including the correction for the impact of temperature on water density and the final conversion from water pressure to water depth are all accomplished carried out online in real time.

Unlike the low-cost depth sensor, the HACH submerged probe is set to scan and log the water level every minute, and field personnel were required to collect the data each week due to the logger’s internal memory capacity. This has resulted in hours of data missing in some weeks of this field sensor comparison test. Furthermore, these differences in logging methods for each sensor means the datasets are mismatched (i.e., the number of total measurements are non-identical). To make a direct comparison between the low-cost sensor and the HACH probe, the HACH depth measurements were matched into the low-cost sensor dataset with a maximum of 1 min time difference tolerance. The HACH data logger’s internal timer was verified with the standard internet clock multiple times throughout the experiment, and the time differences were always less than a minute. Hence, the level measurement difference due to time difference is negligible.

**Data analyses.** Similar to our laboratory study (Section 3.1.1), we estimated the absolute error of the new sensor for each measurement point (Equation (4)), but this time *d_true_* was replaced by the HACH depth measurement assuming negligible uncertainty. Similarly, percentage errors and relative uncertainties were estimated (Equations (5) and (6)).

There were some cases where air pressure readings were not available; for example, during our experiment, the air reference sensor stopped logging four times due to battery issues, which resulted in missing air pressure data. We excluded data from the analysis that did not have air pressure readings available within 30 min of depth measurement.

#### 3.1.3. Accounting for Depth Sensor Drift

As reported above, we calibrated the offset between depth and air sensor at the start of both our lab and field experiments (this offset is used in Equation (1) to estimate water depth). Initial results demonstrated sensor drifting and that this offset would require some re-calibration if the data were to be used to accurately measure absolute water depths. As such, in this paper, we use the datasets to help determine how often this offset is required to be re-calibrated and whether an automatic calibration process could be employed. Furthermore, we explore the potential to ignore some of this systematic drift by using trend detection (i.e., detecting rates of change instead of absolute depth). 

**One calibration before installation.** To allow a comparison to other drift correction methods, this first option was to simply assume no re-calibration of probes after installation. Hence, the offset calculated at the start of the lab and field experiments were assumed to be constant throughout the entire experiment. Under this assumption, we calculated the absolute errors, percentage errors and the relative uncertainty exactly as that described in the above sections (i.e., using Equations (4)–(6)).

**Figure 3:**
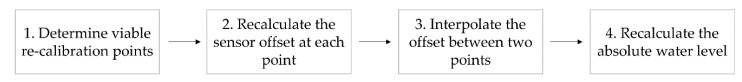
The processes of manual and automated sensor re-calibration.

**Manual re-calibration.** We used the lab study to assess manual re-calibration efficiency. The re-calibration was undertaken based on the four steps shown in Figure 3. Firstly, the SWITCH sensors were determined to be re-calibrated each time when they were taken out from the water column and placed in air. In total, each sensor was removed from water 7 times with an average of 6 days per re-calibration. Secondly, the offset (*e_cal_* of Equation (1)) was calculated by using the sensor pressure minus the air reference pressure at each re-calibration point. Thirdly, between two re-calibration points, the pressure offset at each timestep was estimated using linear interpolation by assuming the drifting rate was constant. Finally, the absolute water level was re-calculated based on the interpolated pressure offset by using Equation (1) at each timestamp. 

Once the above manipulations were made, the absolute differences, percentage errors and the relative uncertainties were re-calculated (following Equations (4)–(6)) by using the data collected during the entire test period (6-day re-calibration frequency on average) and a 14-day period between two re-calibration points; the results were used to compare with only one sensor calibration before the start of the test.

**Automated re-calibration.** We used the field study to determine whether an automatic re-calibration process could be used to obtain accurate absolute water depth measurements. The concept here is like the laboratory test; when the low-cost sensor is out of water, we re-calibrate the difference between the air and the depth sensor. Unfortunately, this field system was very rarely dry, and since the HACH probe was 93 mm above the invert, we could only determine when depths dropped below 102 mm above the invert of the pipe (i.e., the exact depth was unknown when water levels dipped below 102 mm). As such, we re-calibrated our low-cost depth sensor when water levels were 102 mm (when HACH measured 9 mm water depth), but this time instead of re-calibrating the offset to equal the difference in the air and the depth probes, we re-calibrated the offset to also account for the water pressure that exists between the HACH and the low-cost depth sensors. In summary, we conducted this by following four steps (Figure 3), noting that all these steps can be done automatically on remote servers.

**Figure A2:**
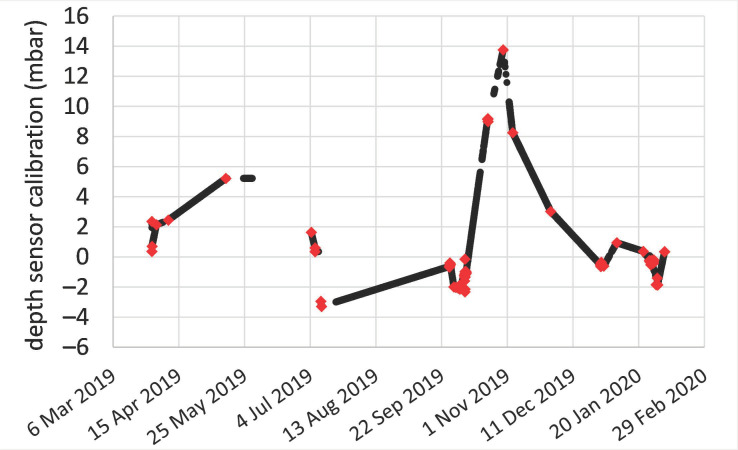
Depth sensor calibration coefficients throughout the monitoring period; red dot—Automatic sensor re-calibration when the HACH probe at its lowest detection limit 9 mm; black dashed line—The linear interpolated calibration coefficient between two automatically generated calibration point; the calibration coefficient is assumed to be constant for the period before the sensor dead.

Firstly, when the HACH’s raw measurement was precisely equal to 9 mm (i.e., 102 mm true water depth), the time points were recorded as viable re-calibration points. In total, 69 re-calibration points occurred on 32 different days. Secondly, based on the sensor installation condition (Figure 2b), the low-cost depth sensor should read exactly 85 mm at the re-calibration points. The offset for each of the 69 re-calibration points was then calculated using Equation (1)—see Figure A2 for the result of the re-calibration offset. Thirdly, the pressure offset at each timestep was estimated using linear interpolation assuming the drifting rate was constant between two re-calibration points. Finally, at each timestamp, the absolute water depth was re-calculated based on the linear interpolated offset.

Once the above adjustments were made, the absolute differences, percentage errors and relative uncertainties were re-calculated (following the same equations as above) to compare with the results obtained from only one calibration before installation.

**Trend detection.** When the purpose of water sensing is to detect the presence of a flow event inside the water network (i.e., a spike of water depth), it is common to use anomaly detection algorithms that rely only on the rate of change of water parameters. For example, the rate of water depth, *S_i_*, could be calculated and used for anomaly detection:

(7) $$s_{i}=\frac{∆d}{∆t}=\frac{d_{i}-d_{i-1}}{t_{i}-t_{i-1}}$$

where Δ*d* and Δ*t* represent the depth and time difference between two measurements, here we used the field study to understand whether re-calibration of the depth sensor was compulsory to obtain accurate trend detection. Using Equation (7), the first derivative of the water depth was calculated for the low-cost sensors and the HACH probe. Since the low-cost sensors and the HACH probe two sensors had different scan and logging intervals (HACH—one scan and log every minute; low-cost scan every 10 s to calculate and log minutely averages), hourly averaged water depth was used in the trend calculation to reduce the impacts of these differences. The errors, percentage errors and relative uncertainties were then calculated between the low-cost sensors and the HACH probe (Equations (4)–(6)). This process was repeated twice: one using the sensor dataset when only one calibration before installation was conducted and another using the adjusted sensor dataset obtained when using the automated re-calibration dataset.

### 3.2. Conductivity Sensor

#### 3.2.1. Lab Testing

The proposed low-cost EC sensor was tested and calibrated in the lab with eight EC standard solutions ranging from 0 to 55 mS/cm. A portable EC meter (HI98192 from Hanna Instruments, Woonsocket, RI, USA, [34]), capable of measuring EC between 0 and 400 mS/cm with 1% uncertainty, was used to determine reference EC concentrations. For sensor calibration, both HI98192 m and the low-cost sensors recorded ten EC and temperature readings after the measured temperature was stabilised. The averaged EC levels were used to generate a linear calibration curve for future field deployment. Absolute errors, percentage errors and relative uncertainty were estimated based on similar equations as Equations (4)–(6). The resolution of the low-cost sensor was also estimated using the range of true EC level divided by the range of raw analogue readings.

#### 3.2.2. Field Comparison

Four low-cost water conductivity sensors were first calibrated in the lab and then installed along an urban creek at four different locations (Site A–D). Since this short creek is close to the bay, the upstream sensors at Site A and B were expected to have low EC levels, while the downstream two sensors at Site C and D, where very close to the seashore tend to experience higher EC readings due to the seawater input. As such, the EC sensor could be tested for a broader measurement range at this specific creek than at urban stormwater drains where usually have EC lower than 0.5 mS/cm.

Similar to the depth sensor tested in the field, water EC was scanned every minute but uploaded when the difference between the two scans was more than 10%. Constant power of 5 V was supplied by using one of the digital pins on the Arduino Uno to ensure the EC pins were only powered for one millisecond, minimising the chance of corrosion. Manual conductivity measurements using a calibrated HORIBA water quality meter were obtained once a week for a whole year May 2017 to June 2018). The data were then analysed by correlating the high-end HORIBA sensor results with our low-cost sensor to understand its overall performance. Absolute errors, percentage errors, and relative uncertainty of the low-cost sensor for urban stormwater sensing were also analysed using Equations (4)–(6) (assuming the EC readings of the HORIBA measurement were correct with negligible uncertainty). 

## 4. Results and Discussion

### 4.1. Depth Sensor

#### 4.1.1. Laboratory Study

**Figure 4:**
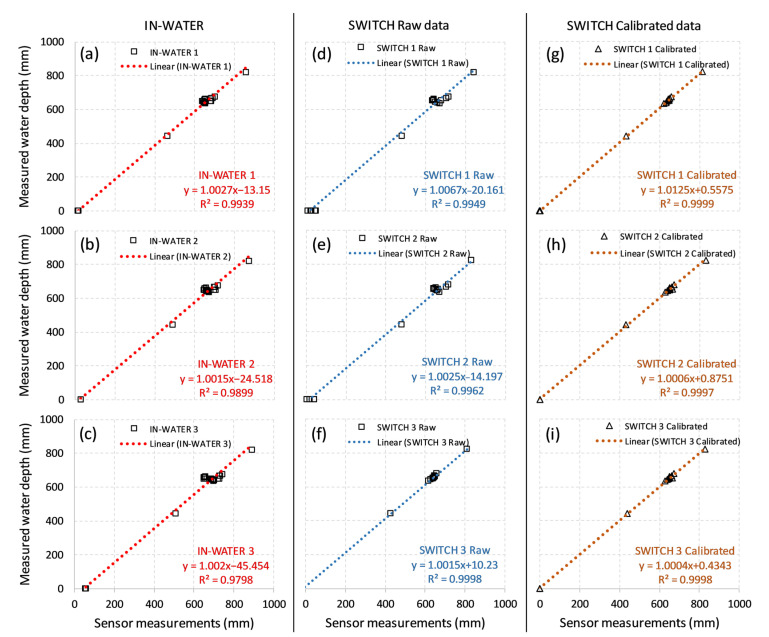
Linear regression curves of water depth between developed low-cost sensors and manual measurements: (**a**) IN-WATER 1, (**b**) IN-WATER 2, and (**c**) IN-WATER 3; (**d**) SWITCH 1, (**e**) SWITCH 2, (**f**) SWITCH 3 (one offset calibration before installation); (**g**–**i**) SWITCH 1–3 sensors after continuous manual re-calibration.

High linear correlations were observed between the true water depths and those recorded by the six different low-cost sensors (Figure 4a–f; R^2^ > 0.98), but the y-intercepts of the linear correlations were off the origin (−45 to 10 mm). This provides some confidence that, over a two-month installation, the depth sensors can measure water depth to some degree of accuracy. 

**Figure 5:**
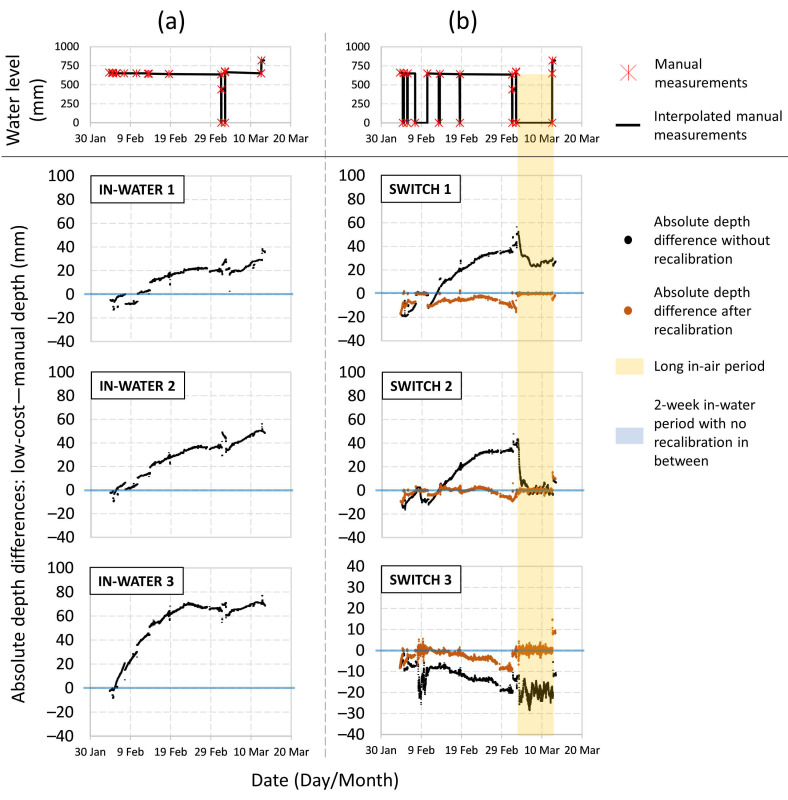
Time series plots of the absolute level difference between low-cost sensor measurements and the interpolated true measurements of water depth for (**a**) IN-WATER 1–3 and (**b**) SWITCH 1–3 sensors; For reference, the true measurement points and interpolated data are shown at the top.

The absolute depth difference between sensor readings and manual measurements for all six examined sensors (Figure 5a) demonstrate that the clear drift occurred after the first day of the lab test started. Indeed, the drift is seen to be more rapid at the beginning of the test and tends to become more stabilised after being placed in water for 20 days. Each IN-WATER sensor experienced different levels of drift over the same period (roughly 20, 40, and 60 mm respectively). The cause of such a significant drift is likely related to the design and manufacturing of MS5803 module (TE Connectivity, Schaffhausen, Switzerland). One possible explanation is the layer of white gel protection on MS5803, which ensures the sensor is waterproofed, might absorb moisture over time, resulting in higher pressure readings with time. 

SWITCH sensors also drifted significantly (black line, Figure 5b). During the first 20 days, SWITCH sensors were moved into air five times. Although the sensor readings were stable in air, they kept drifting once they were placed back into the water column. In fact, after an extended period of air exposure, a large jump in sensor drift and performance was observed for all three SWITCH sensors, somewhat confirming the hypothesis above that moisture uptake in the sensor’s gel could be leading to sensor drift.

**Uncertainties and correcting for sensor drift—One offset calibration, before installation.** Without applying any corrections for sensor drift (i.e., only having one offset calibration without any re-calibration), errors for the IN-WATER sensors were between −1.4 and 69.7 mm (5th and 95th percentiles of all three sensors’ data), percentage error ranges from 0.34% to 10.91% and, by averaging the percentage error, relative uncertainty of the IN-WATER sensors’ depth measurement was 6.17%. For SWITCH sensors, the absolute errors were between −20.6 and 34.4 mm (5th and 95th percentiles of all three sensors’ data), the percentage error ranges from 0.72% to 5.49% and the relative uncertainty was 3.01%.

**Uncertainties and correcting for sensor drift—Manual re-calibration.** By manually re-calibrating the offset of SWITCH sensors every time they were taken out of the water, a marked improvement was observed between manually measured water depth and sensor data (Figure 4g–i: R^2^ > 0.999 and y-intercept < ±1 mm and Figure 5b: orange lines). Manual calibration resulted in absolute errors that were ranging between −8.4 and +2.3 mm, percentage errors between 0.05% and 1.48%, and the relative uncertainty of 0.58%. These significant reductions in errors and uncertainties clearly demonstrate the power of the manual re-calibration process and the measurement sensitivity achievable by this new sensor.

Table 1 compares the error of low-cost sensors under different offset re-calibration frequency. Results indicate that by manually re-calibrating the low-cost sensor every two weeks, the absolute error should be within a ±10 mm range with a relative uncertainty of less than 1%. More frequent re-calibration frequencies (e.g., a 6-day average of the lab test) are not recommended for field deployment since sensor performance was not significantly improved compared with re-calibration every two weeks.

#### 4.1.2. Field Deployment

**Figure 6:**
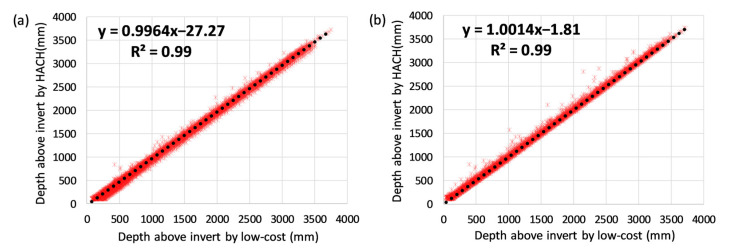
Comparing the depth of water above the drain’s invert from the low-cost depth sensor and the HACH submerged probe: (**a**) raw low-cost sensor readings without conduction continuous automatic re-calibration; (**b**) low-cost sensor conducting re-calibration when HACH reached its lowest detection limit—9 mm.

In the field, the low-cost depth sensor was able to detect water levels of between 50 mm and 3.5 m. The sensor adequately monitored the true water level in the stormwater drain, as demonstrated by the high linear correlation with the HACH probe over the entire test (Figure 6a: R^2^ = 0.99). Similar to what has been found in the lab study, the low-cost sensor overestimated true water level in the urban drainage network (negative y-intercept in the linear correlation curve).

**Figure 7:**
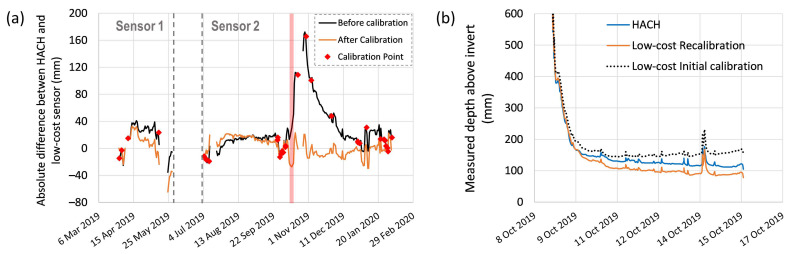
(**a**) averaged daily difference between low-cost water depth sensor and HACH submerged probe for both low-cost Sensor 1 and Sensor 2—The positive difference represents the low-cost water depth is higher than that of HACH probe; (**b**) the measured water depth of low-cost Sensor 2 and HACH submerged probe during the red-shaded period.

To further understand the sensor’s performance under field conditions, daily averaged absolute differences between the low-cost and HACH probe is presented (Figure 7a: black line). For most of the test, both low-cost sensors (Sensor 1 and 2) demonstrated an absolute difference within a range of ±40 mm. However, it is also noticeable that Sensor 2 experienced a significant spike in drift between October and December 2019. Measured water depths from both HACH and low-cost sensors after a large rain event (the red-shaded period) are also plotted (Figure 7b). While the HACH measured baseflow level kept decreasing as what to be expected after a rain event, the low-cost sensor measurements detected an increasing trend in baseflow (black dashed line).

**Figure 8:**
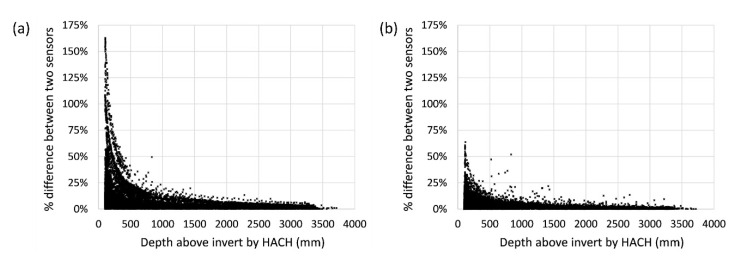
The percentage error between the low-cost and HACH sensors at different water levels: (**a**) low-cost sensor measurements with one offset calibration only; and (**b**) low-cost data with automated sensor re-calibration.

**Uncertainties and correcting for sensor drift—One offset calibration before installation.** Without any sensor drift correction, the absolute difference between the low-cost and HACH sensors ranged from −18.1 to 110.8 mm (5th and 95th percentiles), the percentage errors were between 0.44% and 39.23%, and the relative uncertainty was 10.65%. As with all depth sensing methods [35], it was expected that both low-cost and HACH sensors have higher relative uncertainties when measuring smaller depths as compared to higher depths. As such, the percentage error is more variable at lower depths as compared to higher depths (Figure 8a).

**Uncertainties and correcting for sensor drift—Automated re-calibration.** Automatic re-calibration improved the sensor’s performance significantly (Figure 6b: R^2^ > 0.99 and y-intercept = −1.81), and the large drift spike was fully corrected (Figure 7a). The re-calibration resulted in absolute sensor errors between −36.7 and 33.8 mm (5th and 95th percentiles), percentage errors between 0.20% and 16.15% (significantly reduced across the measuring range compared with only one calibration, Figure 8b) and a relative uncertainty of 4.88%. The success of the automatic re-calibration in this field test relied on the high re-calibration frequency (on average, the low-cost sensor was re-calibrated every 9 days). The higher uncertainties observed for the field test, as compared to the lab test where re-calibration was done every 14 days, are likely because: (1) the lab test used true water depths measured manually by a ruler to calculate uncertainties, yet this field test relied on another sensor with its own inherent uncertainties to calculate errors, (2) field deployments are less controlled than laboratory systems and hence introduce naturally more potential for uncertainty in measurements (e.g., high speed flow and sediments).

**Figure 9:**
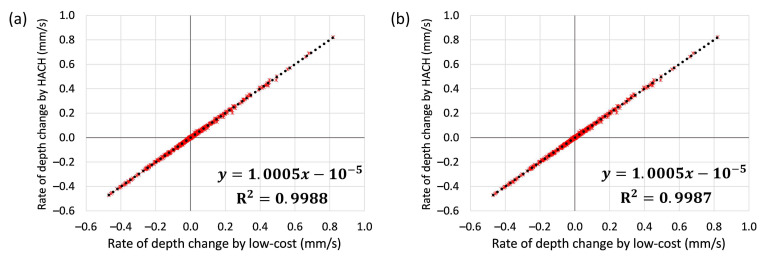
Comparing the rate of depth change (trend) between low-cost and HACH probes: (**a**) with only one offset calibration before installation; and (**b**) by using the low-cost sensor data after automated re-calibration.

**Uncertainties and correcting for sensor drift—Trend detection.** Based on the field test, the detected trend of water depth by the low-cost sensor was also compared with HACH. By only having one pressure offset calibration before the installation, Figure 9 indicates that the low-cost sensor can adequately detect the water level trend (intercept was 0.00, the slope was 1.00 and scatter around the 1:1 line was almost non-existent). The absolute sensor error was extremely small at ±0.002 mm/s (5th and 95th percentiles), suggesting sensors without any calibration can be confidently used to detect rates of change and depth trends.

#### 4.1.3. Application of the Low-Cost Depth Sensors

The results of both lab and field tests suggest the low-cost depth sensor can adequately measure water level and detect trends in either a standing water body or stormwater drains with flowing water. Manual and automatic sensor re-calibration significantly improved its performance by correcting the drifting issue. Here, we further explore and discuss the usage of the developed low-cost sensors for different distributed real-time sensing applications in urban stormwater networks.

**Trends-only.** In some applications, including illicit discharge detection, the absolute water depth is only part of the required information and, in fact, the change in depth, or rather, the trend thereof is of most importance. For example, detecting whether unexpected water flow exists does not require absolute depth measurements to be known, instead only whether trends can be measured. The field test suggests that our low-cost sensors do not require any manual or automatic re-calibration to adequately monitor trends in water level fluctuations in the drainage network. If the purpose of monitoring does not require the measurement of absolute water levels, then systematic sensor drift can be ignored without any data correction or manipulation. As such, the sensor’s maintenance frequency will only be determined by the potential battery life of the module, which will significantly reduce the cost of management in the field.

More importantly, the trend data can be continuously calculated on the web server and an alarm can be triggered when an unexpected flow event is detected by the sensor. This enables the authority’s field crew with a higher chance to locate the source of illicit discharges even the flow event is transitory or occurred during afterhours period, which were the main reasons why the previous illicit detection program was failed [9,10,11].

**Automated re-calibration**. In some applications such as monitoring a stormwater drain, it might be able to automatically re-calibrate the offset between the air sensor and the depth sensor if it is possible to estimate when the sensor sits out of water or is known to be below a certain depth. For example, the two EC pins of our low-cost sensor are 6 mm above the invert of the pipe if it is installed flat on the bottom of the pipe. This design provides a chance for automatic sensor re-calibration: as when EC module turns from positive readings to zero or vice versa (it reads absolutely zero when it is in air), we can be certain that the absolute water level in the drain at that specific time will be roughly 6 mm. By doing that, the sensor’s accuracy can be significantly improved without any further field work requirements. The saved money can help with increasing the total number of sites of a distributed sensing network without breaking the limited budget. Therefore, for monitoring purpose like future stormwater model development that requires better understanding of the flow dynamic within one catchment [4,36], more than 10 developed low-cost sensors can be installed at different locations by spending the same amount of money as installing one high-end sensing station at the catchment outlet.

One potential limitation of the automatic sensor re-calibration in our current design by using the fixed position of EC pins is that the sensor has no knowledge about the absolute water level when it is as shallow (depths < 6 mm). If the drain is completely dry, the low-cost sensor may detect a negative absolute water depth. In the lab test, between 3 March and 12 March, SWITCH sensors were placed in air for a 10-day period (yellow shaded region in Figure 5b). The accumulated sensor drift during the inundation period could be partially removed (SWITCH 1) or even completely removed (SWITCH 2), but the automatic offset re-calibration was unable to take this information into consideration to correct the sensor drift by averaging pressure readings in the air as was done for manual re-calibration. Hence, the use of automatic re-calibration approach requires more field studies and under different flow conditions.

**Manual adjustment.** If the above options are not possible, then manual calibration must be performed at least every 2 weeks if accurate absolute depth sensing results are expected. This includes cases such as monitoring of water level in a constructed wetland or a rainwater storage tank, where the water level will rarely reach as low as the position of the EC pins. Ideally, the sensor’s manual re-calibration process should not be undertaken by lifting the sensor out of water, as it might cause the sensor’s level of drift to quickly change in the air, thereby affecting the accuracy of linear interpolated sensor offsets. Instead, the low-cost sensor can be re-calibrated with a water level gauge, to back-calculate the pressure offset as has been done for the automatic field sensor re-calibration in Section 3.1.3.

The current 14-day sensor re-calibration frequency is a drawback comparing with high-end sensors for the water level monitoring of major stormwater assets (e.g., wetland, ponds, and rainwater tanks). The MS5803 module’s drift issue suggests that current off-the-shelf, pressure-based, and low-cost water level sensors do not yet have the long-term durability as their high-end counterparts. Although ultrasonic/radar based water level sensor has already proved its accuracy for large asset monitoring [23,24], it will still be easier to have a pressure-based sensor in the water to monitor the level together with other quality parameters in one setting. The future research of low-cost water depth sensing should focus on reducing the cost of current high-end sensor chips.

### 4.2. Conductivity Sensor

#### 4.2.1. Lab Testing

**Figure 10:**
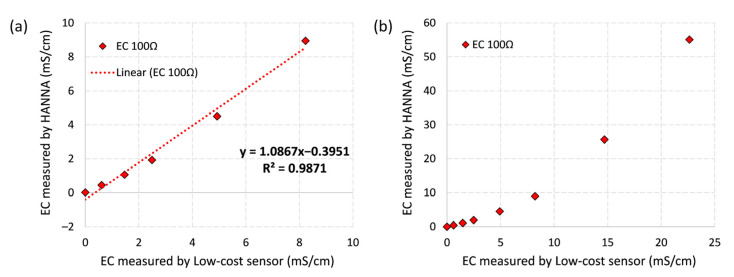
(**a**) correlation between the low-cost conductivity sensor and HANNA’s measurement results in standard EC solutions ranging from 0 to10 mS/cm; and (**b**) correlation in solutions between 0 and 60 mS/cm.

The calibration result between the low-cost EC sensor and HANNA meter is shown below in Figure 10a. The low-cost sensor tends to have a strong linear correlation relationship with the HANNA when the measurement range is between 0 and 10 mS/cm (R^2^ = 0.98). The low-cost sensor’s precision is 0.021 mS/cm (i.e., 0.021 mS/cm per one analogue unit), which is sufficient for urban drainage monitoring due to its relatively low EC levels between 0.1 and 1 mS/cm [37]. Absolute errors of the low-cost sensor were between −0.41 mS/cm and 0.46 mS/cm (min and max), percentage errors ranged from 4.53% to 39.58%, with a relative uncertainty of 17.53%.

When higher level of EC solutions (>10 mS/cm) were considered in the lab sensor calibration analysis (Figure 10b), the low-cost sensor tended to have a shifted linear relationship with HANNA between 10 and 60 mS/cm. Sensor precision at this range is roughly 0.24 mS/cm per analogue unit, which is significantly lower than that of between 0 and 10 mS/cm.

#### 4.2.2. Field Comparison

**Figure 11:**
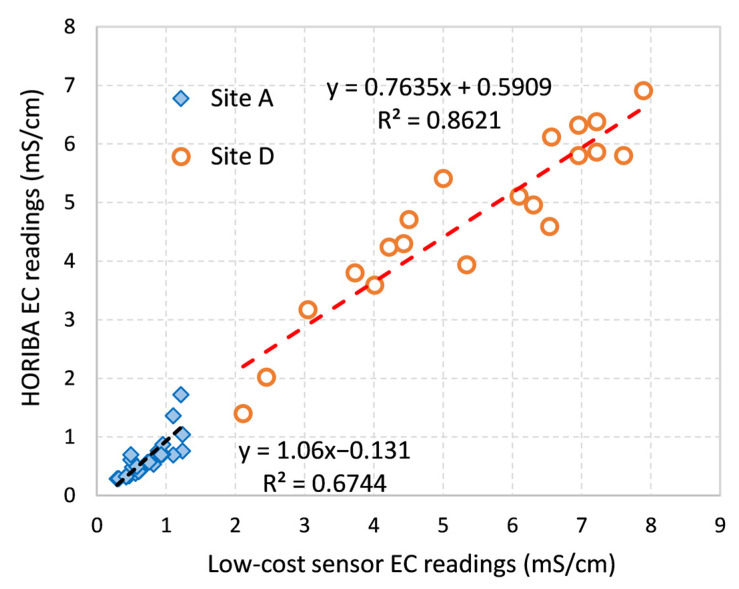
Low-cost sensor field readings compared with HORIBA measurement at Site A and D.

**Figure A3:**
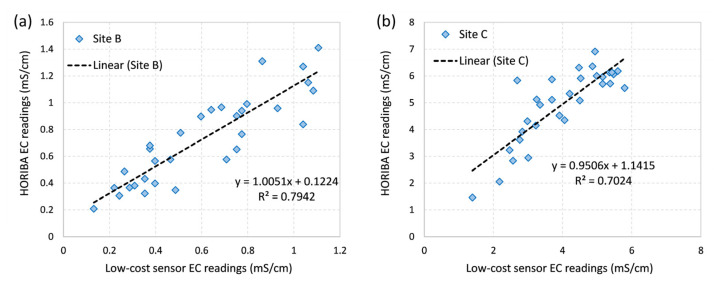
(**a**) The correlation between low-cost EC sensor readings and HORIBA measurement at Site B; (**b**) the correlation comparison at Site C.

Table 2 shows the calibration curve of each sensor generated based on standard EC solutions between 0 and 10 mS/cm in the lab before the field deployment. An appropriate degree of linearity was obtained for all four sensors and they were similar to each other. The field EC measurement results (Figure 11) demonstrated that Site A’s EC level was between 0.1 and 2 mS/cm whereas Site D had higher EC readings ranging from 1 to 7 mS/cm due to seawater input. The highly linear correlation between the low-cost sensor readings and HORIBA measurements at each site (Figure 11 and Figure A3) indicate that trends in urban stormwater EC levels can be adequately monitored just by two small stainless-steel rods with a simple voltage divider circuit design.

Table 3 below shows the range of EC levels, absolute sensor errors, percentage errors and the relative uncertainty of each low-cost sensor tested in the field. In terms of the absolute error, low-cost sensors at Site A and B demonstrated smaller error range, while Site C’s sensor significantly underestimated the EC level and Site D’s sensor overestimated the water conductivity. For the percentage errors, all four sensors tested in the field demonstrated similar performance as the lab calibration results.

#### 4.2.3. Conductivity Sensor’s Applications

The usage of the low-cost EC sensor is determined based on the background EC level of the environment. For water sources with EC levels below 10 mS/cm, such as stormwater, rainwater storage systems, lakes, ponds, and other natural waterways, the low-cost sensor can be used to measure the absolute EC level with an accuracy of ±0.5 mS/cm and with a relative uncertainty of 18%. One potential issue of the low-cost sensor is that its calibration curve has a negative y-intercept (between −0.2 and −0.4 mS/cm from the lab and field tests), which will result in negative EC levels when the low-cost sensor’s measurement is around 0.2 to 0.4 mS/cm. A calibration curve with an intercept at the origin could solve potential negative readings, but at the cost of the sensor’s accuracy (reduced to ±0.6 mS/cm, higher relative uncertainty of 26%).

The sensor’s performance for measuring EC levels above 10 mS/cm was not studied in this paper. Another individual calibration curve needs to be generated between 10 and 60 mS/cm range. For water sources including industrial discharges and urban sewage, the level of conductivity can be highly variable and significantly higher than other natural water sources. In these cases, although this EC sensor is not highly accurate as high-end sensors, it still provides valuable information on how poor the water quality is for a cost of USD 1.

Furthermore, as discussed for the automatic re-calibration function of the depth sensor, the EC sensor module also forms an essential component to be as a permanent water level gauge of the low-cost water sensor design in this paper. For the concept of distributed sensing with low-cost and low-power sensors, the high cost of labour for site maintenance (i.e., sensor cleaning, battery changing, and sensor replacement) combined with many sites in each project will result in a large spend, which is controversial to the initial concept of distributed sensing. By integrating the EC module together with the depth sensor, we are not only (1) correcting the depth sensor’s drifting issue, (2) measuring the water EC as an important water quality parameter, but also (3) significantly reduce the number of site visits required to ensure the distributed sensing is achievable for urban water monitoring especially the illicit discharge detection.

## 5. Conclusions

The lack of low-cost stormwater monitoring techniques was recognised as a limitation of the current development of spatially distributed stormwater sensing, and the quick detection of illicit discharges in urban drainage network. This study presents the first integrated water depth, EC, and temperature sensor design based on off-the-shelf products, which is low-cost (costing USD 25) and easy to install (three water parameters measured by one sensor, instead of three). Whilst the depth sensor experienced a drift issue, and it did not affect the sensor’s performance in detecting the trend of water level (i.e., when using it for illicit discharge detections). To adequately monitor absolute water level (i.e., accuracy ±10 mm), the sensor needed to be manually re-calibrated at least every two weeks, or automatically re-calibrated by the conductivity sensor module that was integrated in the all-in-one sensor design. The automatic re-calibration algorithm enables the sensor to be only maintained when the battery is flat, thereby significantly reducing the cost of sensing network management. Apart from being a depth sensor’s calibrator, the EC sensor also adequately monitored absolute water conductivity between 0 and 10 mS/cm. Overall, this study presented an integrated and low-cost stormwater sensor that is capable for vast deployment to achieve the distributed sensing future, and also can be used in other water sources such as marine and wastewater.

## Figures and Tables

**Table 1:** The absolute error, percentage error and the relative uncertainty of all three SWITCH sensors under different sensor re-calibration frequency (i.e., no re-calibration, 14 days, and average 6 days).

Re-Calibration Frequency	SWITCH 1			SWITCH 2			SWITCH 3		
	Absolute Difference (mm) (5th, 95th Percentiles)	Percentage Error (%) (5th, 95th Percentiles)	Relative Uncertainty (%)	Absolute Difference (mm) (5th, 95th Percentiles)	Percentage Error (%) (5th, 95th Percentiles)	Relative Uncertainty (%)	Absolute Difference (mm) (5th, 95th Percentiles)	Percentage Error (%) (5th, 95th Percentiles)	Relative Uncertainty (%)
None	(−14.81, 36.99)	(0.69, 5.69)	3.59	(−8.33, 34.03)	(0.58, 5.36)	3.39	(−23.03, −6.46)	(0.96, 3.03)	1.80
14-day	(−9.77, −2.42)	(0.38, 1.54)	0.82	(−6.00, 2.53)	(0.03, 0.94)	0.35	(−8.77, −1.82)	(0.28, 1.38)	0.77
Average 6-day	(−11.10, −2.45)	(0.36, 1.82)	1.00	(−6.37, 2.84)	(0.03, 1.30)	0.41	(−8.51, 0.63)	(0.05, 1.34)	0.56

**Table 2:** The resistance setup and calibration curves for the deployed sensors in the field.

Site	Resistance	EC = a × Measured EC + b (R^2^)
A	100 Ω	EC = 1.0963 × measured EC − 0.2331 (0.9959)
B	100 Ω	EC = 1.1075 × measured EC − 0.2004 (0.9963)
C	100 Ω	EC = 1.2226 × measured EC − 0.2685 (0.9983)
D	100 Ω	EC = 1.3023 × measured EC − 0.2338 (0.9990)

**Table 3:** Each site’s low-cost sensor’s absolute errors, percentage errors and the relative uncertainty by comparing with the HORIBA.

Site	HORIBA Measured EC Range (mS/cm)	Absolute Difference (mS/cm) (5th, 95th Percentiles)	Percentage Error (%) (5th, 95th Percentiles)	Relative Uncertainty (%)
A	(0.29, 1.72)	(−0.51, 0.44)	(4.54, 60.40)	31.12
B	(0.28, 1.34)	(−0.35, 0.16)	(0.41, 45.00)	23.21
C	(1.84, 6.55)	(−2.51, 0.17)	(3.69, 42.96)	18.65
D	(1.43, 6.88)	(−0.40, 1.94)	(0.52, 50.42)	17.42

## Data Availability

The data presented in this study are openly available in [A Low-Cost Water Depth and Electrical Conductivity Sensor for Detecting Inputs into Urban Stormwater Networks—research data] at [10.17632/kjtt3ws3y2.1].

## Appendix A

Figure A1 below shows the four major steps of waterproofing the proposed sensor into the sensor case. The first step requires the base of the 3D printed sensor cover to be placed on a flat bench (Figure A1a). The second step is to vertically lower the sensor board into the chamber of the sensor case (Figure A1b). Two EC pins need to be pushed into the screw terminal on the sensor board through two holes on the front wall of the sensor case. A H-shape holder is required to be placed on top of the sensor, which is designed to hold the sensor down with pressure. The third step is to slide the sensor case’s lid into the case’s base (Figure A1c). Some hot glues are recommended to be placed around the sensor cable before the lid is in place. The final step is to inject the potting compound gel from the small hole next to the sensor cable (Figure A1d). The sensor case is then slowly filled up by the gel from the bottom which ensures the sensor is highly waterproofed from all angles and suitable for long-term deployment in the drainage system.
